# An Uncommon Presentation of Polyarticular Septic Arthritis Following an Atraumatic Treadmill Run: A Case Report

**DOI:** 10.7759/cureus.114434

**Published:** 2026-08-12

**Authors:** Karen Garza, Namra Khan, Hana Shah, Emily Dickinson, Carina Rose Lorenzen, Lorena Bonilla

**Affiliations:** 1 Department of Translational Medicine, Florida International University, Herbert Wertheim College of Medicine, Miami, USA; 2 Department of Family Medicine, Baptist Health South Florida, Miami, USA

**Keywords:** atopic dermatitis, avascular necrosis, corticosteroids, osteomyelitis, septic polyarthritis

## Abstract

Septic arthritis is an urgent orthopedic condition typically caused by gram-positive bacteria, such as *Staphylococcus aureus*. It poses a risk for complications like osteomyelitis, osteonecrosis, sepsis, and chronic pain. In this case report, we present a 33-year-old woman with a history of atopic dermatitis, obesity (body mass index 40 kg/m^2^), and hypertension, who presented to the emergency room with fever and right ankle pain following a jog on the treadmill. Initially, she was diagnosed with plantar fasciitis, for which she was prescribed corticosteroids. Her condition rapidly progressed, and she was ultimately diagnosed with polyarticular septic arthritis and admitted for treatment. Her condition was further complicated by deep vein thrombosis, avascular necrosis, and osteomyelitis of the right lower extremity. Despite initial treatment with antibiotics and surgical intervention, her symptoms persisted, leading to repeat surgery, readmission, and chronic mobility problems.

In this article, we present a case of atraumatic onset of septic arthritis after exercise in a young patient without classic immunosuppression. This case underscores the importance of considering a diagnosis of septic arthritis in patients with overlapping risk factors such as atopic dermatitis, obesity, and corticosteroid use. It illustrates the benefits of advanced imaging for accurate, prompt assessment and intervention. The condition's early presentation can mimic other inflammatory disorders, complicating diagnosis. This case report contributes to the growing but limited body of literature that associates septic arthritis with risk factors such as atopic dermatitis, obesity, and corticosteroid use.

## Introduction

Septic arthritis is a rheumatological disorder typically caused by gram-positive organisms, most commonly *Staphylococcus aureus*, followed by *Streptococcus pneumoniae*, in adults. Due to the risk of joint damage secondary to bacterial invasion, host inflammation, and tissue ischemia, septic arthritis is considered an orthopedic emergency. Individuals with pre-existing joint disease, skin conditions, obesity, or those on immunosuppressive medications may have increased susceptibility for developing septic arthritis [1,2]. Septic arthritis typically occurs following bacterial invasion of the joint synovium via direct injury like a puncture wound, hematogenous seeding from a systemic infection, or contiguous spread from adjacent osteomyelitis. It typically presents as an acute onset of pain in a single joint, most commonly the knee, hip, shoulder, or ankle, and is accompanied by fever, swelling, and reluctance to move the affected joint [1]. Polyarticular septic arthritis is less common and is more often seen in a patient with underlying rheumatoid arthritis or corticosteroid use. It is of particular clinical importance, as it can be associated with a mortality rate of up to 32%, even with appropriate therapy, as compared to a mortality rate of less than 5% in monoarticular septic arthritis [3]. Diagnosis of septic arthritis is made via joint aspirate and culture analysis, supported by elevated C-reactive protein (CRP) and erythrocyte sedimentation rate (ESR). Prompt treatment with antibiotics, with or without joint drainage, is aimed at limiting infection and further joint damage [1].

## Case presentation

A 33-year-old woman with a medical history of atopic dermatitis, obesity (body mass index 40 kg/m^2^), and hypertension presented to the Emergency Department (ED) for right ankle pain localized over the right lateral malleolus. Her ankle pain started after jogging on the treadmill, though she denied a specific injury. Within hours of the run, she was unable to bear weight due to the severity of the pain. She developed fever and chills during this time and was found to have a temperature of 39.1°C in the ED. Radiographs in the ED showed no evidence of acute fracture or traumatic malalignment. Thus, she was discharged home with nonsteroidal anti-inflammatory (NSAID) medications and was recommended to follow up with podiatry. Figure 1 shows radiographs of the right lower extremity during this ED visit. 

**Figure 1:**
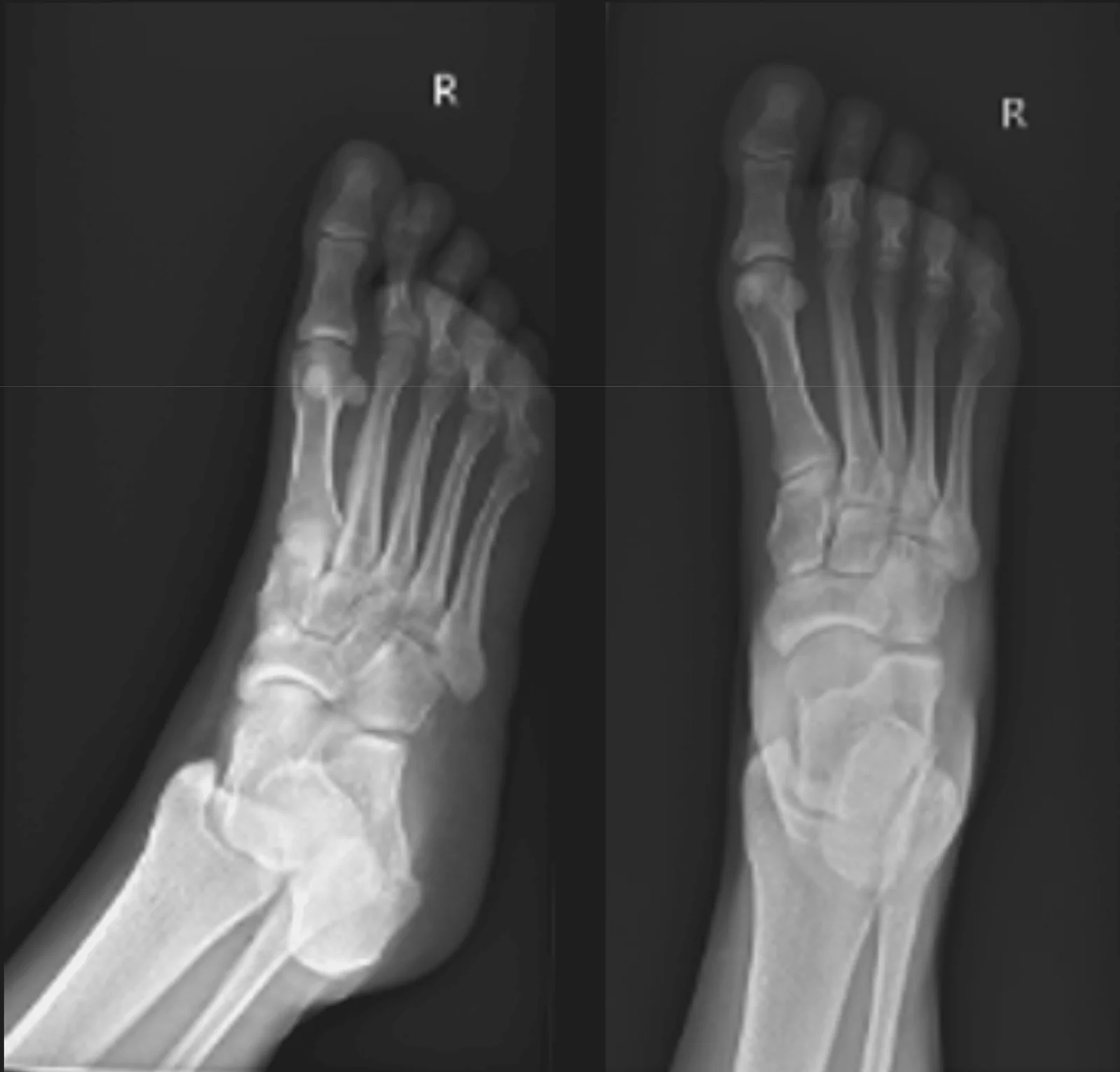
Two-view radiograph of the right lower extremity obtained on day 1.

The following day, she returned to the same ED with persistent pain and was again provided reassurance and discharged home. On the third day, she was evaluated by a podiatrist who diagnosed her with plantar fasciitis and prescribed her a course of oral methylprednisolone. Her right lower extremity was placed in an air cast, and she was made non-weight-bearing. In the following three days, the patient developed significant swelling of her right lower extremity.

On day nine, the patient returned to the ED for a third time. She presented with continued lower extremity complaints in addition to new-onset left shoulder pain, with decreased range of motion. On examination of the right lower extremity, there was swelling distal to the knee, with erythema localized to the lateral and dorsal foot. Distal pulses were palpable only with Doppler ultrasound. Her passive and active range of motion was limited in all planes secondary to pain. Her skin was warm, with blistering noted proximal to the digits of the foot and posterior to the lateral malleolus, though crepitus was not found. Left shoulder exam revealed mild swelling and erythema; it was tender only on active abduction. Distal pulses and sensation of the left upper extremity were fully intact. Figures 2, 3 are clinical images from this examination.

**Figure 2:**
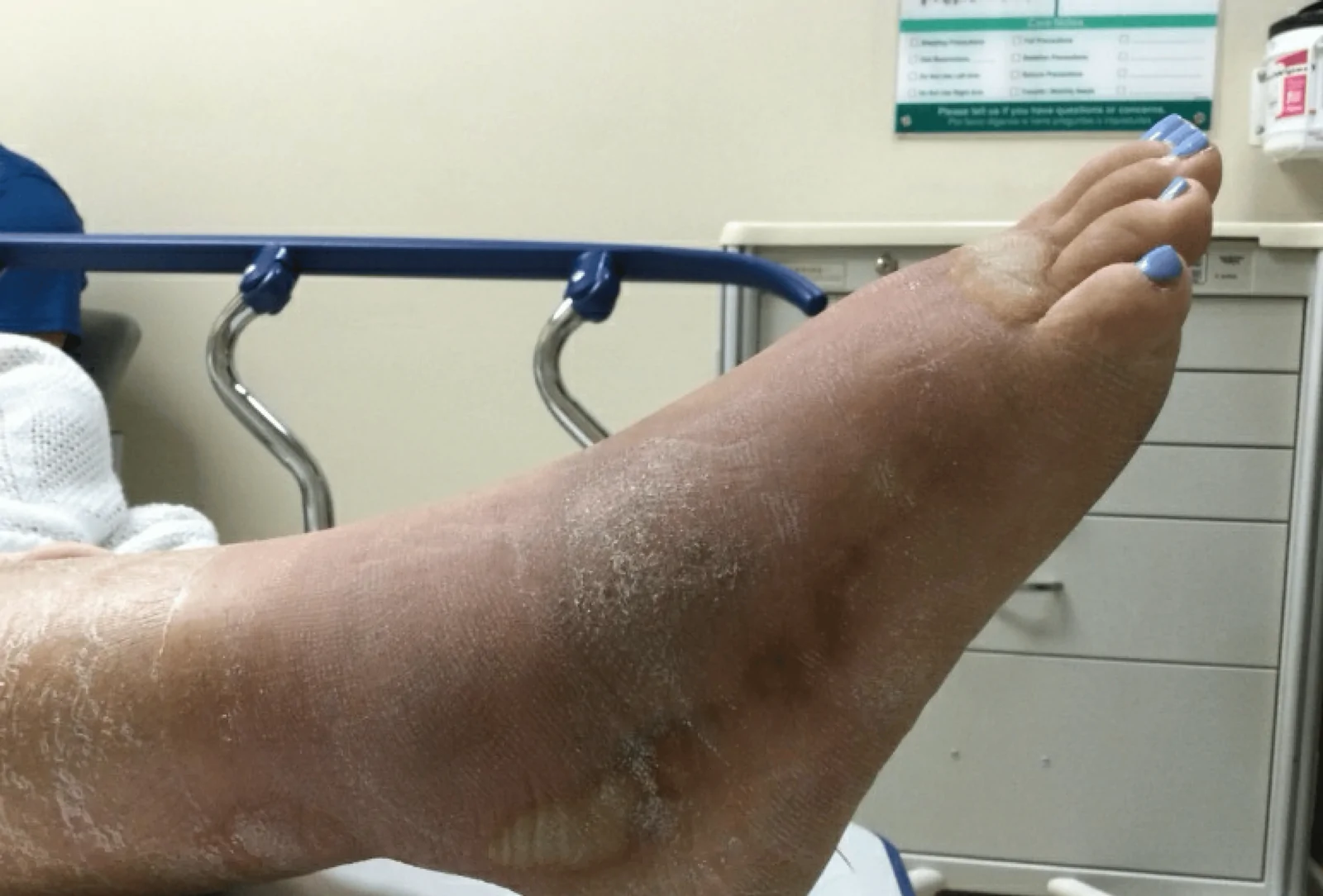
Right lower extremity on day 9.

**Figure 3:**
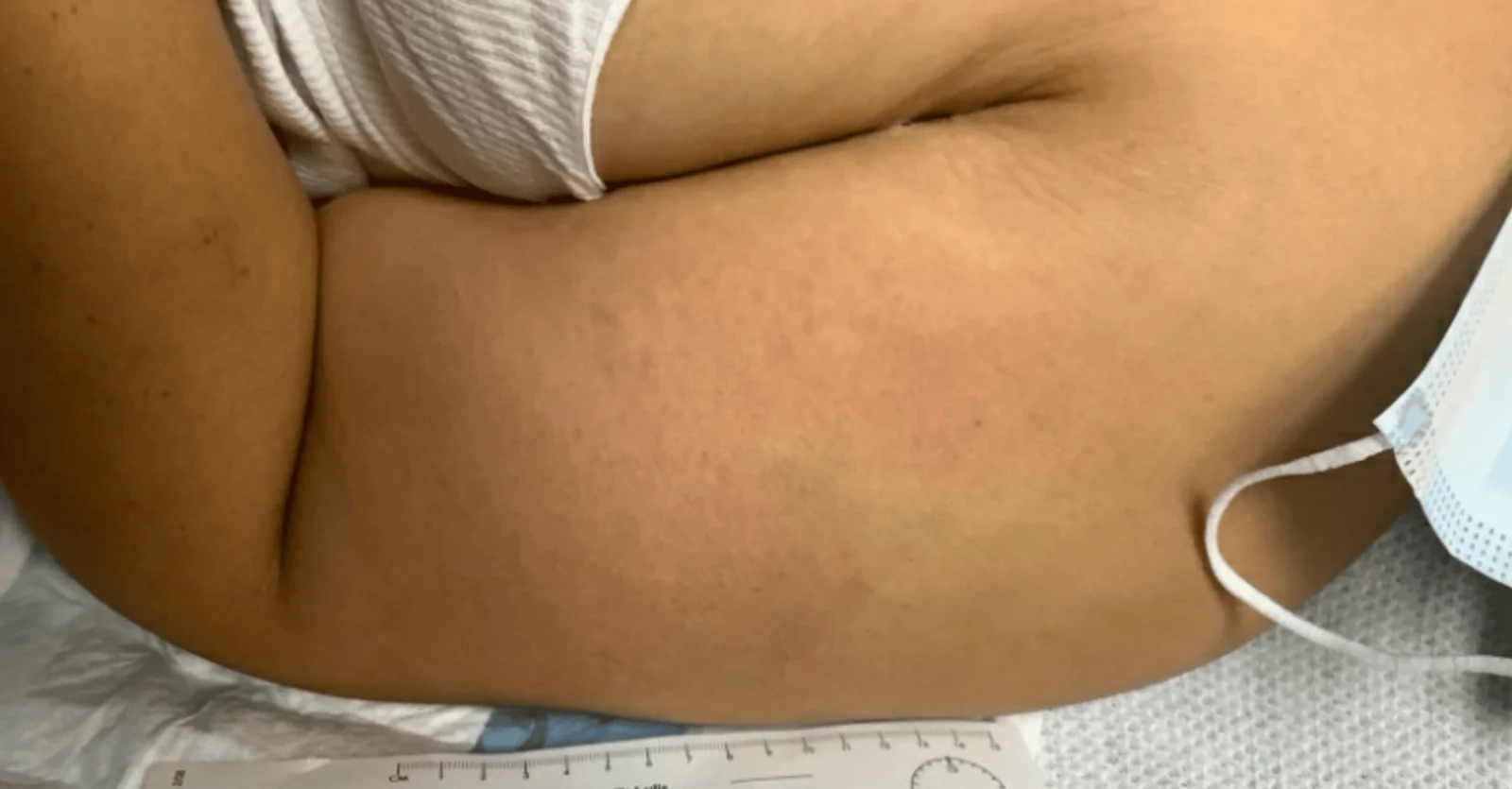
Left upper extremity on day 9.

The patient was afebrile at this time (37.6°C), but laboratory values were remarkable for leukocytosis, with a white blood cell (WBC) count of 35.6 × 10³/µL (neutrophil-predominant), CRP of 298 mg/L, and ESR of 107. While the WBC count might have been impacted by the systemic steroids, the obvious inflammatory process and accompanying elevations in CRP and ESR suggest that the WBC count was at least in part reflective of a true infectious process. Pertinent findings are summarized in Table 1. 

**Table 1:** Vitals and laboratory values on day 9. *Reference range reported for females under 40 years of age.

Variables	Result	Reference Range
Temperature	37.6	36.5-37.5 (°C)
While blood cells (WBC)	35.62	4.5-11.0 (× 10^3^ cells/mm^3^)
C-reactive protein (CRP)	298	0.08–3.1 (mg/L)
Erythrocyte sedimentation rate (ESR)*	107	≤20 (mm/hr)

Additionally, a Doppler study of the right lower extremity showed deep vein thrombosis (DVT) involving the peroneal and posterior tibial veins. Radiographs of the right lower extremity revealed swelling overlying the lateral malleolus that was not present on prior imaging. Radiographs of the left shoulder were unremarkable. The patient was admitted to the medical inpatient service for evaluation and treatment of polyarticular septic arthritis and DVT. Figure 4 shows the right lower extremity dopplers, followed by Figure 5, which shows the new lateral malleolus swelling. 

**Figure 4:**
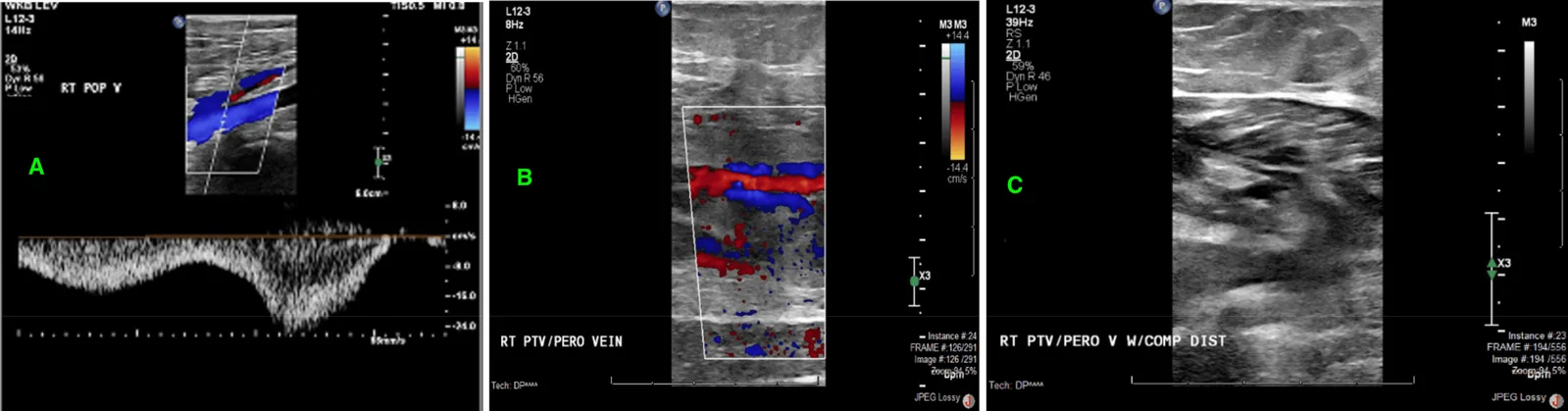
FIGURE 4: Duplex ultrasound of the right lower extremity. From left to right: (A) Spectral Doppler of the popliteal vein demonstrating normal phasic flow, consistent with patency. (B) Color Doppler of the posterior tibial and peroneal veins demonstrating absent intraluminal flow, suspicious for thrombosis. (C) Grayscale compression ultrasound of the same veins demonstrating incomplete venous coaptation, confirming deep vein thrombosis.

**Figure 5:**
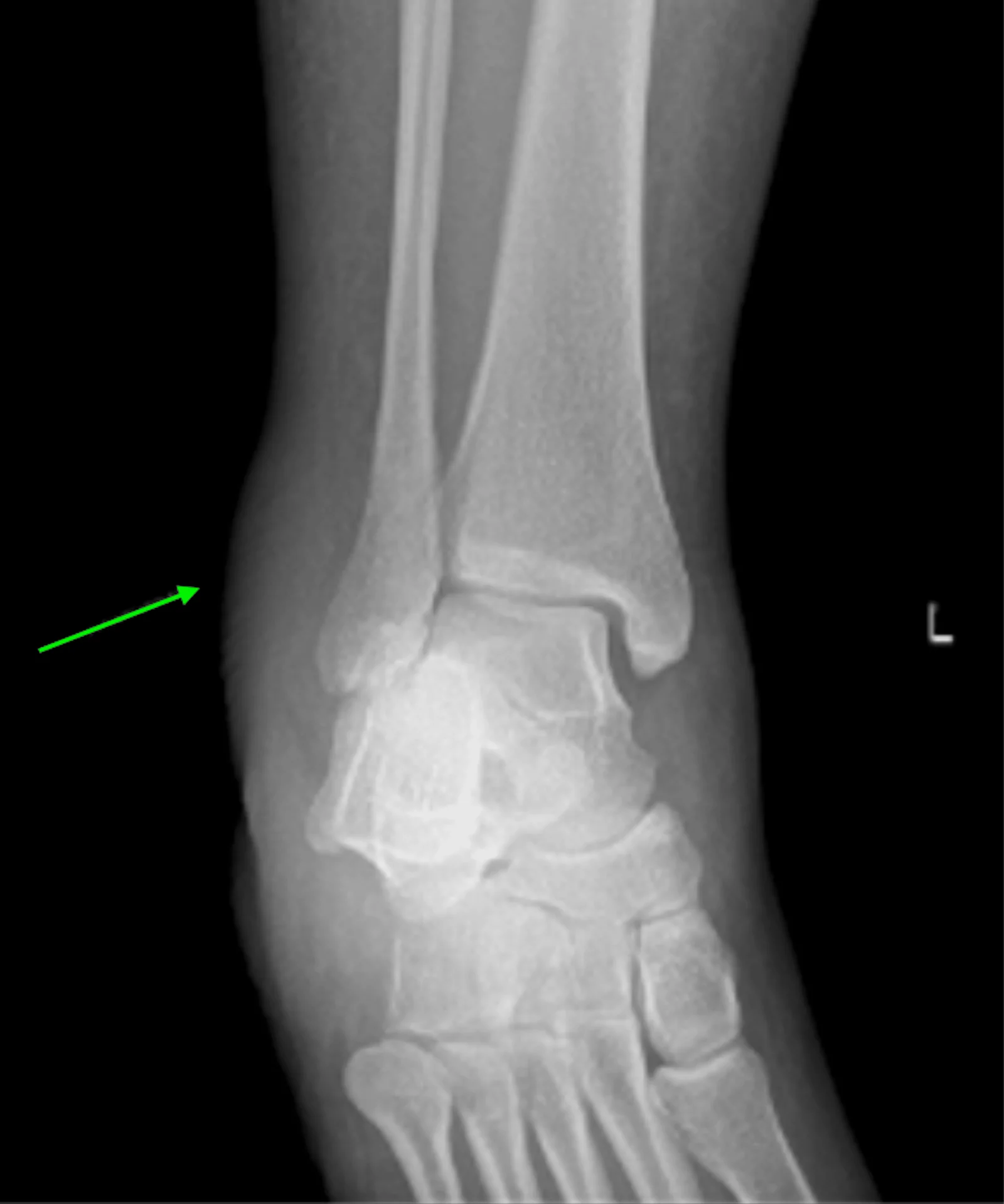
Radiograph showing right malleolus soft tissue swelling on day 9.

On day 10, a right lower extremity computed tomography (CT) scan showed a joint effusion suggestive of septic arthritis, with diffuse fat stranding of subcutaneous tissue likely related to cellulitis. There was also a small fluid collection within the muscular fascia that likely represented a small abscess. A CT scan of the left shoulder similarly showed a joint effusion concerning for septic arthritis. The patient refused magnetic resonance imaging (MRI) at this time. She was evaluated by an orthopedic surgeon, who recommended surgical intervention out of concern for polyarticular septic arthritis and possible necrotizing fasciitis. Empiric treatment with intravenous daptomycin, ceftriaxone, and clindamycin was initiated. Figure 6 shows a fluid collection seen on CT of the lower third of the right leg.

**Figure 6:**
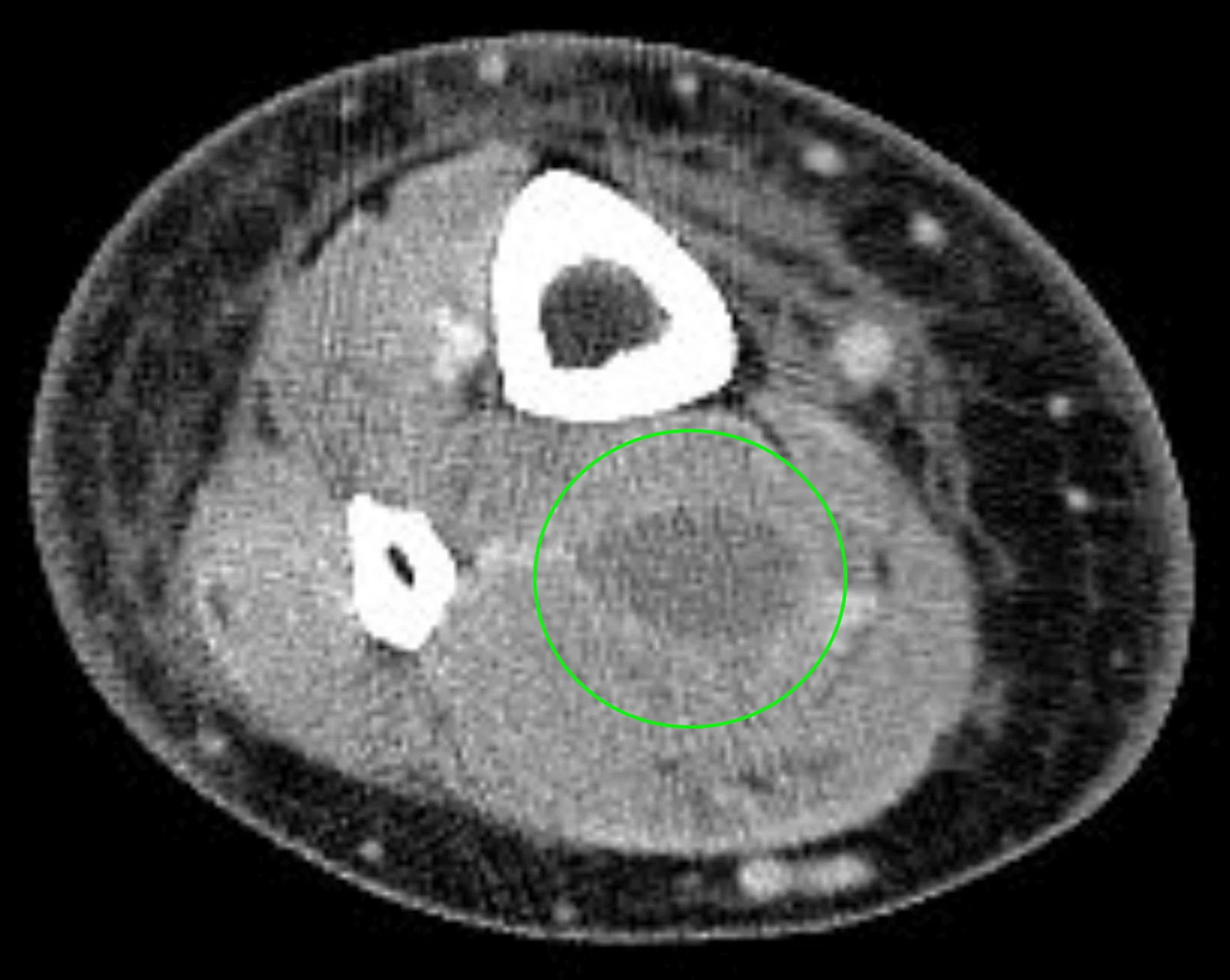
Right lower extremity CT scan showing 19.3 mm × 12.7 mm area likely representing a small abscess.

On day 11, the patient underwent arthrotomy of the affected joints, with abscess incision and drainage (I&D) of the dorsolateral aspect of the right ankle and dorsal aspect of the right foot at the level of the third metatarsal shaft. Aspirates of both sample areas and blood cultures grew *Streptococcus pyogenes,* and her antibiotics were switched accordingly to cefazolin. Figure 7 is a clinical image from two days after this procedure.

**Figure 7:**
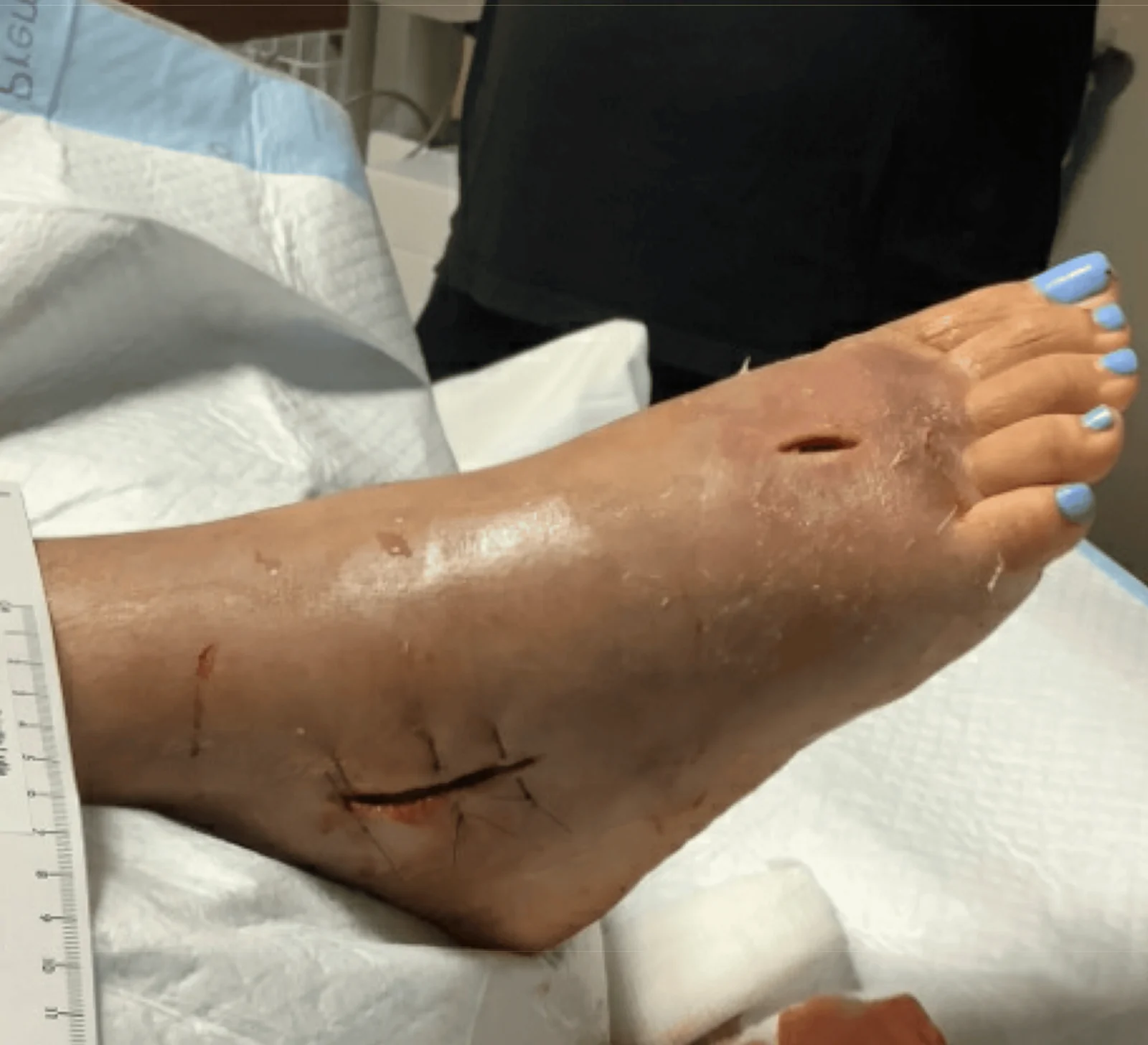
Right lower extremity on day 13, two days after arthrotomy and incision and drainage procedures.

The patient continued to complain of severe postoperative pain in her right lower extremity, such that on day 16, she ultimately required a sedated MRI of the right tibia, fibula, and foot. Imaging revealed no evidence of acute osteomyelitis; however, there was evidence of avascular necrosis (AVN) of the talar head and an enhancing tibiotalar joint effusion, suggesting persistent septic arthritis. Additionally, there was extensive enhancing cellulitis, myositis, and multiple abscesses. Figure 8 shows a longitudinal tubular rim-enhancing fluid collection spanning the entire posterior calf at the level of the posterior tibialis muscle.

**Figure 8:**
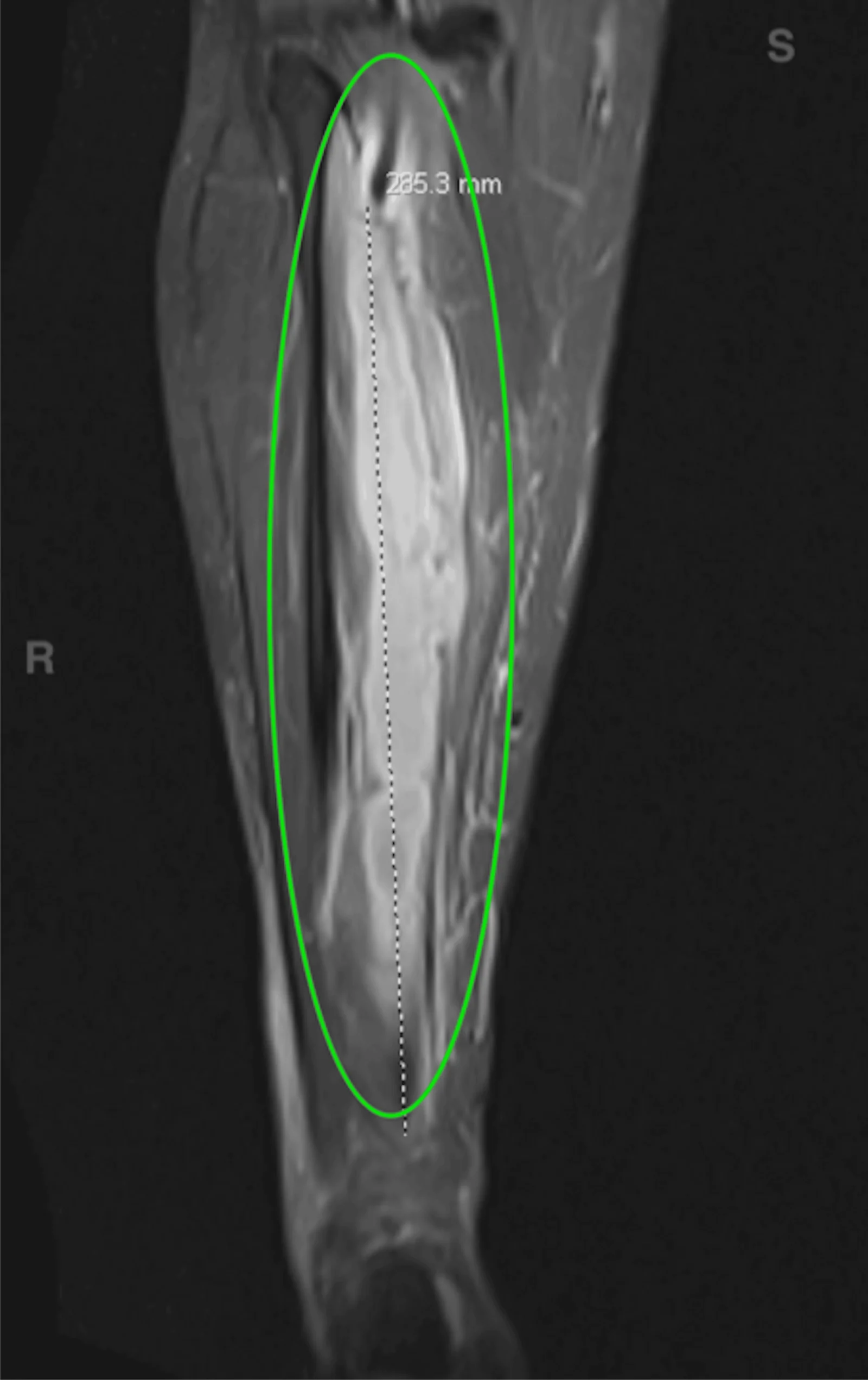
Right tibia/fibula MRI on day 16 showing a tubular rim-enhancing fluid collection spanning the posterior calf.

MRI of the right foot showed extensive circumferential enhancing subcutaneous and intramuscular edema, rim-enhancing 1.1 cm fluid collection immediately plantar to the proximal shaft of the third metatarsal bone, an ill-defined fluid collection in the dorsal aspect of the midfoot measuring 1.8 cm, and a third fluid collection within the intrinsic plantar musculature at the level of the calcaneus measuring 1.3 cm. Figure 9 shows the 1.1 cm collection plantar to the third metatarsal bone. 

**Figure 9:**
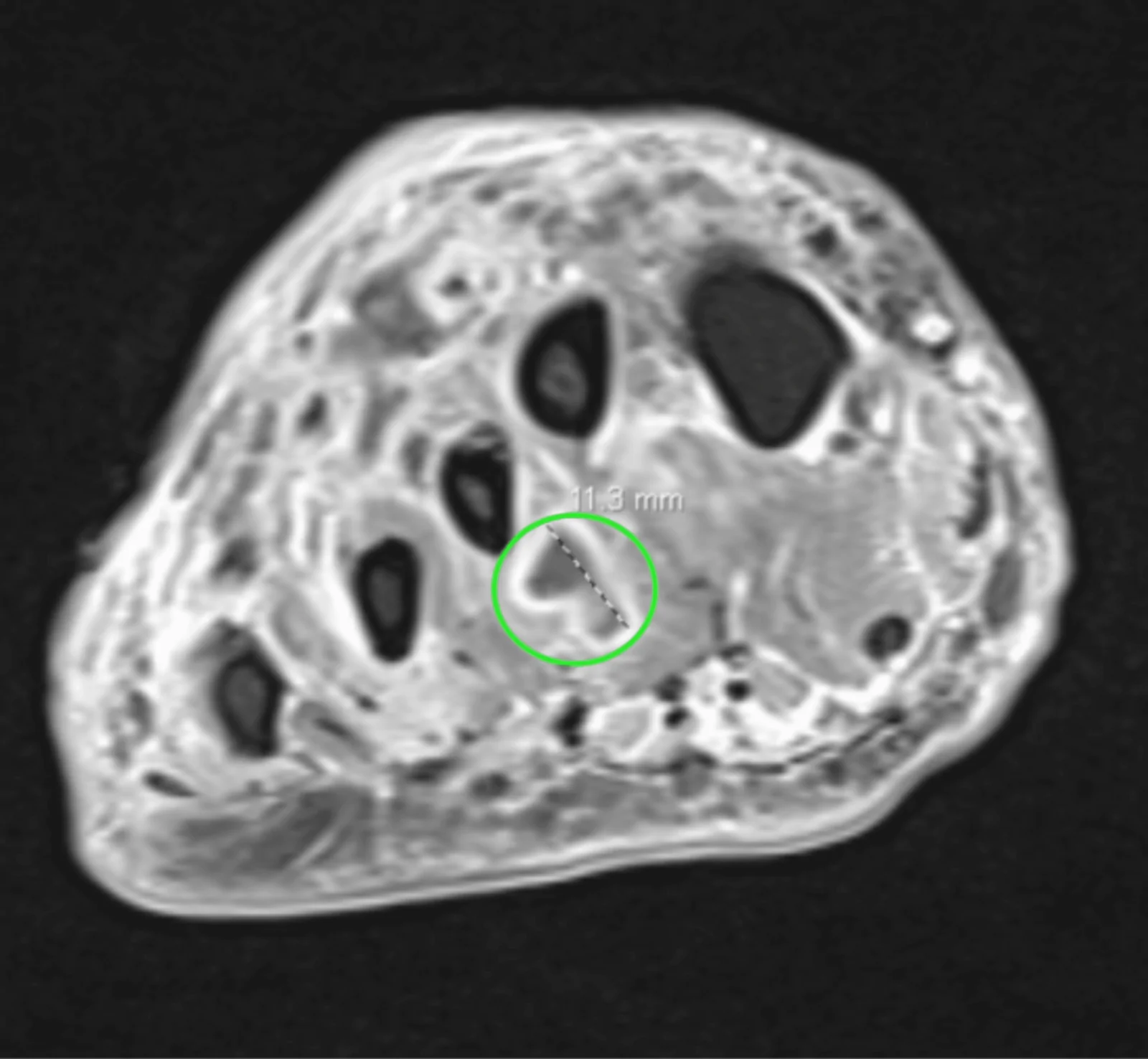
Right foot MRI on day 16 showing rim-enhancing 1.1 cm fluid collection immediately plantar to the proximal shaft of the third metatarsal bone.

The patient underwent additional ankle arthrotomy, I&D of the right ankle, and right foot abscess. Furthermore, she had right leg I&D of the deep posterior compartment abscess with placement of two Hemovac drains. Cultures from this operation were negative. The transesophageal echocardiogram was negative for vegetations. She was maintained on intravenous (IV) antibiotics and non-weight bearing status. 

She was discharged home on day 24 after a two-week hospitalization. Upon discharge, IV cefazolin was replaced with one gram oral cefadroxil every 12 hours for an additional three weeks. Home health care and outpatient physical therapy referrals were provided. Figure 10 summarizes the patient’s clinical course from the onset of her symptoms to her discharge.

**Figure 10:**
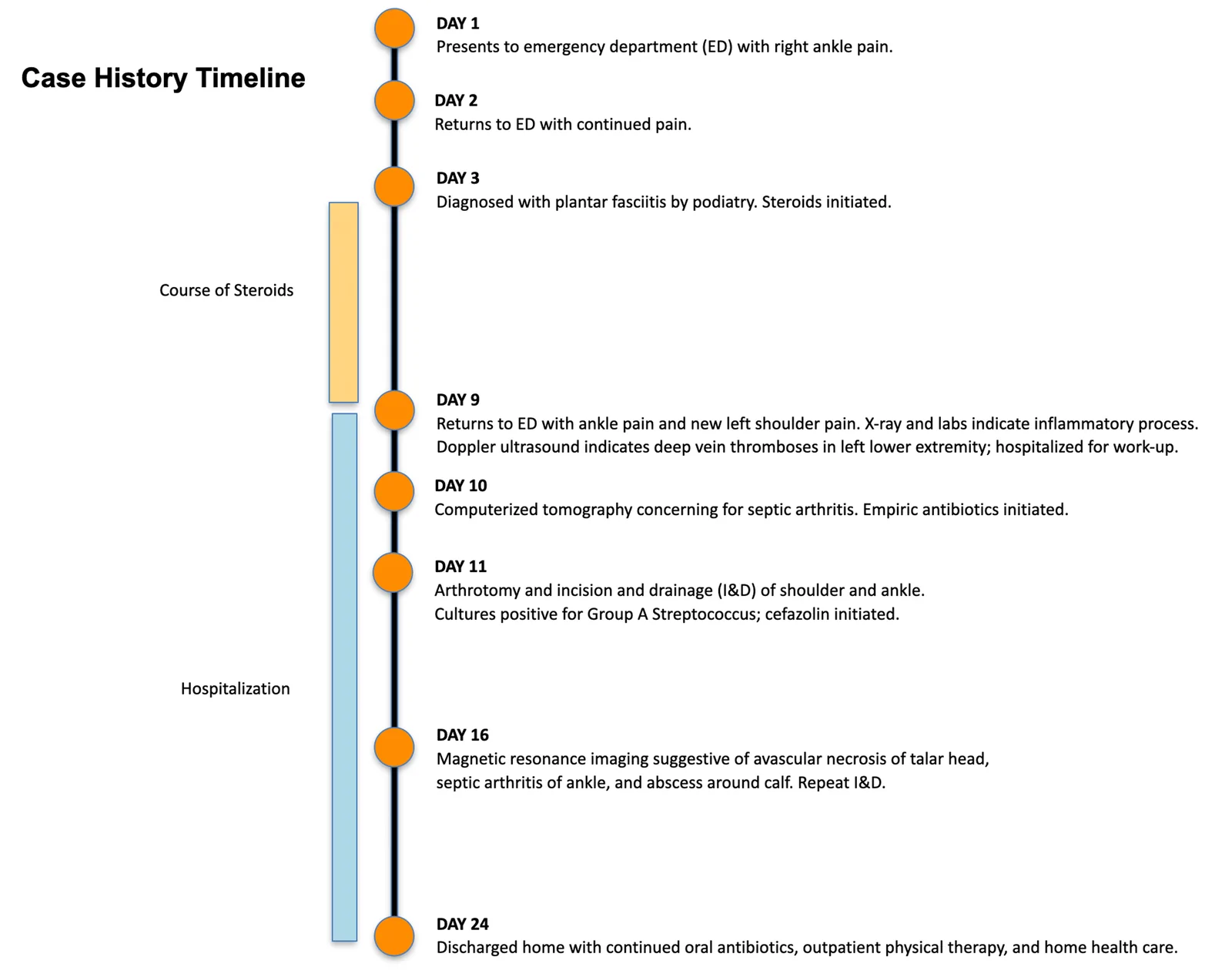
Timeline of patient’s initial clinical course.

Follow-up and outcomes

The patient returned to the hospital 12 days after her discharge per the recommendation of her podiatrist, who noted persistent right foot equinus deformity and significant pain with foot and ankle movement. On this readmission to the hospital, the patient was hemodynamically stable and afebrile. The WBC count was normal at 10.62 × 10³/µL. Bone biopsy revealed osteomyelitis of the right tibia, talus, and cuboid bone. The patient was treated conservatively; she was maintained non-weight-bearing and on antibiotics. She was discharged nine days later on cefazolin, 2 g IV every eight hours for six weeks. Subsequent bone biopsies showed resolution of the osteomyelitis. With conservative management and physical therapy, she regained 50% of her range of motion. Ten months after her initial admission, she was still partially weight-bearing. 

## Discussion

This case exemplifies the importance of diagnosing and managing septic arthritis in a patient with multiple overlapping risk factors, including atopic dermatitis, obesity, and corticosteroid use, and highlights the severe sequelae of delayed diagnosis. Septic arthritis, particularly in its polyarticular form, can be difficult to diagnose early, as it often mimics other inflammatory or rheumatologic disorders. Despite the delayed diagnosis of septic arthritis in this patient, the rapid initiation of appropriate antibiotics and surgical management upon diagnosis likely prevented life-threatening complications such as sepsis and septic shock.

Several factors may have predisposed this patient to polyarticular septic arthritis, most notably a compromised skin barrier as a result of atopic dermatitis. Ogonowska et al. highlight that patients with atopic dermatitis are at increased risk of bacterial infections due to a compromised skin barrier, which can lead to colonization by pathogens such as *Staphylococcus aureus*. This colonization can serve as a portal for septic arthritis, especially in patients with poorly controlled eczema. Additionally, atopic dermatitis is associated with an impaired immune response, which can increase the risk of infections and worsen outcomes [4]. A 2015 review in the *Journal of Investigational Allergology & Clinical Immunology* summarized published case reports linking atopic dermatitis with septic arthritis and osteomyelitis. The review noted that large joints, particularly the hip and knee, were most frequently affected, with Staphylococcus aureus as the predominant pathogen [5]. More recently, a 2020 review highlighted additional cases of osteo-articular infections associated with atopic dermatitis reported between 1988 and 2017 [6].

Obesity was another significant contributor to this patient’s clinical deterioration due to both immune system dysfunction and mechanical factors. Chronic low-grade inflammation associated with obesity, driven by cytokines like IL-6 and TNF-α, compromises both the innate and adaptive immune responses, thereby weakening the body’s ability to combat infections [7]. Neutrophils and macrophages, critical for pathogen clearance, have reduced functionality in obese individuals, further impairing infection control [7]. A large cohort study demonstrated that obese individuals have an increased risk of hospitalization due to skin and soft tissue infections (adjusted hazard ratio (aHR) 2.46), osteomyelitis (aHR 1.70), and necrotizing fasciitis (aHR 3.54) [2]. Moreover, structural changes induced by excess adipose tissue can promote bacterial overgrowth and increase susceptibility to skin and soft tissue infections, including cellulitis and abscess formation [7]. 

Furthermore, the patient’s treatment with corticosteroids may have compounded the risk of septic arthritis, given that even a short course of oral methylprednisolone has been shown to increase infection risk [8]. Systemic corticosteroids, though beneficial in managing inflammatory conditions, also carry a risk of infection secondary to immunosuppression, depending on the dose and duration [9]. Studies included in a meta-analysis, which primarily focused on pediatric populations, suggested that when used alongside antibiotics, corticosteroids could reduce the duration of symptoms and inflammatory markers in septic arthritis without any significant adverse effects [10]. The use of corticosteroids in these cases highlights the need to strike a balance between managing inflammation and mitigating the risk of severe infections, reinforcing the need for careful monitoring when prescribing these medications. 

The association between inflammation and coagulopathy is also important in understanding this patient’s clinical complications. Autoimmune skin disorders, such as atopic dermatitis, can activate coagulation pathways via local and systemic inflammatory responses [11]. In our patient, underlying atopic dermatitis may have increased her risk of both septic arthritis and coagulopathy, manifesting as DVTs. Alternatively, the patient’s DVTs might have been a direct consequence of the septic arthritis. Septic arthritis, particularly when it involves multiple joints, can provoke a significant inflammatory response. This systemic inflammation, in turn, may activate coagulation pathways, increasing the risk of thrombotic complications. 

The diagnosis of septic arthritis was particularly challenging in this case due to its initial non-specific presentation, which could easily be mistaken for less severe conditions. During the patient’s first inpatient hospitalization, the differential diagnoses to consider included: rheumatoid arthritis, crystal-induced arthropathy, acute fracture, reactive arthritis, Lyme disease, and Pigmented Villonodular Synovitis (PVNS). These conditions share many clinical features with septic arthritis.PVNS, for example, can mimic septic arthritis with pain and joint swelling but requires a different management approach, as it may lead to progressive joint destruction [12]. The patient’s initial presentation with fever, her aforementioned risk factors, and the development of multiarticular involvement necessitated re-evaluation of the initial diagnosis.

Advanced imaging techniques, such as MRI, played a crucial role in the management of this patient. X-rays in the earlier stages of septic arthritis are often unremarkable. Widened joint spaces, bulging of the soft tissues, or subchondral bony changes present only as late findings. MRI is more sensitive in the early stages, revealing abnormalities in surrounding soft tissue and bone, and delineating the extent of cartilaginous involvement [1]. While initial radiographs in this patient did not reveal significant findings, this could not exclude the differential diagnosis of septic arthritis. The patient’s X-rays and CT scans only showed evidence of septic arthritis once the condition was in later stages. The eventual MRI, performed under sedation, excluded osteomyelitis and confirmed the extent of soft tissue involvement, including AVN and multiple abscesses. MRI findings guided surgical decision-making and treatment monitoring. The gold standard for diagnosing septic arthritis is synovial fluid analysis to identify the causative pathogen and guide targeted antibiotic therapy [13]. The combination of joint aspiration, broad-spectrum antibiotics, and possible surgical intervention should be considered promptly, especially in cases with extensive joint and soft tissue involvement or in patients with high-risk profiles for joint destruction or systemic spread of infection.

Despite the complexities involved in the diagnosis and management of this patient, this case report offers valuable insights into the progression of the disease in individuals with overlapping risk factors. A notable strength of this report is that, due to the patient's multiple repeat visits to the same emergency department, we are able to appreciate a detailed timeline of events and the unfortunate sequelae of her uncommon presentation. However, this report is limited in its ability to fully explore the patient's underlying risk factors. Specifically, the lack of detailed information about the patient's history of atopic dermatitis prevents a more comprehensive understanding of whether active eczematous lesions played a pivotal role in predisposing her to septic arthritis. Without this context, it remains challenging to determine the extent to which her skin condition contributed to the disease's progression.

## Conclusions

Maintaining a high index of suspicion in patients with joint pain, fever, and inability to bear weight is crucial in preventing severe complications. In this patient, risk factors including atopic dermatitis and obesity should have further raised the suspicion for the diagnosis of septic arthritis. Atopic dermatitis has the potential to compromise the skin barrier and increase the risk of bacterial infection and hematogenous spread. Thus, septic arthritis should be strongly considered, even when the initial presentation may suggest less severe conditions. The use of imaging modalities, such as MRI, is critical for gauging the extent of infection and guiding medical and surgical management, as radiographs are often unable to detect septic arthritis in its early stages. There is a delicate balance between managing inflammatory conditions with immunosuppressive therapies, such as corticosteroids, and mitigating the risk of infections. This case highlights the need for thorough evaluation and prompt treatment in high-risk populations. 
